# Pressure-adjusted venting eliminates start-up delays and compensates for vertical position of syringe infusion pumps used for microinfusion

**DOI:** 10.1007/s10877-023-01101-6

**Published:** 2023-12-08

**Authors:** Markus Weiss, Pedro David Wendel-Garcia, Vincenzo Cannizzaro, Beate Grass, Philipp Karl Buehler, Maren Kleine-Brueggeney

**Affiliations:** 1 Department of Anesthesia, University Children’s Hospital, Steinwiesstrasse 75, 8032 Zurich, Switzerland; 2 Department of Intensive Care, University Hospital and University of Zurich, Zurich, Switzerland; 3 Department of Neonatology, Newborn Research, University Hospital and University of Zurich, Zurich, Switzerland; 4 Department of Intensive Care, Kantonsspital, Winterthur, Switzerland; 5 Department of Cardiac Anesthesiology and Intensive Care Medicine, Deutsches Herzzentrum Der Charité (DHZC), Berlin, Germany; 6 Charité - Universitätsmedizin Berlin, corporate member of Freie Universität Berlin and Humboldt-Universität Zu Berlin, Berlin, Germany

**Keywords:** Infusion, Syringe, Pump, Start-up, Venting, Central venous pressure, Microinfusion

## Abstract

Microinfusions are commonly used for the administration of catecholamines, but start-up delays pose a problem for reliable and timely drug delivery. Recent findings show that venting of the syringe infusion pump with draining of fluid to ambient pressure before directing the flow towards the central venous catheter does not counteract start-up delays. With the aim to reduce start-up delays, this study compared fluid delivery during start-up of syringe infusion pumps without venting, with ambient pressure venting, and with central venous pressure (CVP)-adjusted venting. Start-up fluid delivery from syringe pumps using a microinfusion of 1 mL/h was assessed by means of liquid flow measurement at 10, 60, 180 and 360 s after opening the stopcock and starting the pump. Assessments were performed using no venting, ambient pressure venting or CVP-adjusted venting, with the pump placed either at zero, − 43 cm or + 43 cm level and exposed to a simulated CVP of 10 mmHg. Measured fluid delivery was closest to the calculated fluid delivery for CVP-adjusted venting (87% to 100% at the different timepoints). The largest deviations were found for ambient pressure venting (− 1151% to + 82%). At 360 s after start-up 72% to 92% of expected fluid volumes were delivered without venting, 46% to 82% with ambient pressure venting and 96% to 99% with CVP-adjusted venting. CVP-adjusted venting demonstrated consistent results across vertical pump placements (p = 0.485), whereas the other methods had significant variances (p < 0.001 for both). In conclusion, CVP-adjusted venting effectively eliminates imprecise drug delivery and start-up delays when using microinfusions.

## Introduction

Delays in start-up fluid delivery are common when using syringe infusion pumps for microinfusion [1, 2]. This is of considerable clinical relevance when starting intravenous inotropic therapy in hemodynamically unstable patients or during syringe changeover in critically ill patients receiving catecholamines [2, 3].

Venting, i.e. pre-running of the syringe infusion pump with draining fluid into a waste channel before directing the drug flow towards the central venous catheter, has been proposed to eliminate start-up delays of the infusion pump [4–7]. Recent data revealed that the venting principle allowed to reduce start-up times, defined as the time to achieve at least 90% of set steady state flow rate, by about 50% when compared to the non-vented approach. However, the shorter start-up times were counteracted by considerable initial backflow into the syringe infusion pump assembly when opening the three-way stopcock leading to comparable start-up fluid delivery using the vented or the non-vented approach [8]. The backflow of fluid from the central venous line into the syringe infusion pump assembly is thought to be caused by the pressure gradient from the central venous pressure (CVP) to the atmospheric pressure in the vented line. Accordingly, we hypothesized that venting of the syringe infusion pump against a pressure equal to CVP will omit this pressure gradient and consecutively avoid fluid shifts into the syringe infusion pump assembly when opening the stopcock [8].

With the clinically relevant aim of eliminating start-up delays of microinfusions and achieving a reliable and predictable drug delivery from the initiation of a microinfusion the aim of this study was to evaluate fluid delivery after start-up of syringe infusion pumps using microinfusions in three situations: without venting, with ambient pressure venting, or with venting adjusted to the central venous pressure.

## Methods

### Experimental setup

Fluid delivery after the start-up of syringe infusion pumps at an infusion rate of 1 mL/h and exposed to a simulated CVP of 10 mmHg was assessed by means of liquid flow measurement. The experiments employed three venting methods: no venting, ambient pressure venting, and CVP-adjusted venting. The pump's position varied: at heart level (zero level), 43 cm below, or 43 cm above this level. The reason to use vertical positions of − 43 cm and + 43 cm was that the distance measured between the syringe outlet of the highest and lowest syringe pump placed in a sevenfold docking station (BD Alaris™ Gateway Workstation 80300UNS-70, CareFusion, Rolle, Switzerland) was 86 cm. Equipment, software and the general experimental setup used for this in-vitro assessment are described in detail elsewhere [8, 9].

### Conducted experiments

#### Standard start-up without venting

**Fig. 1:**
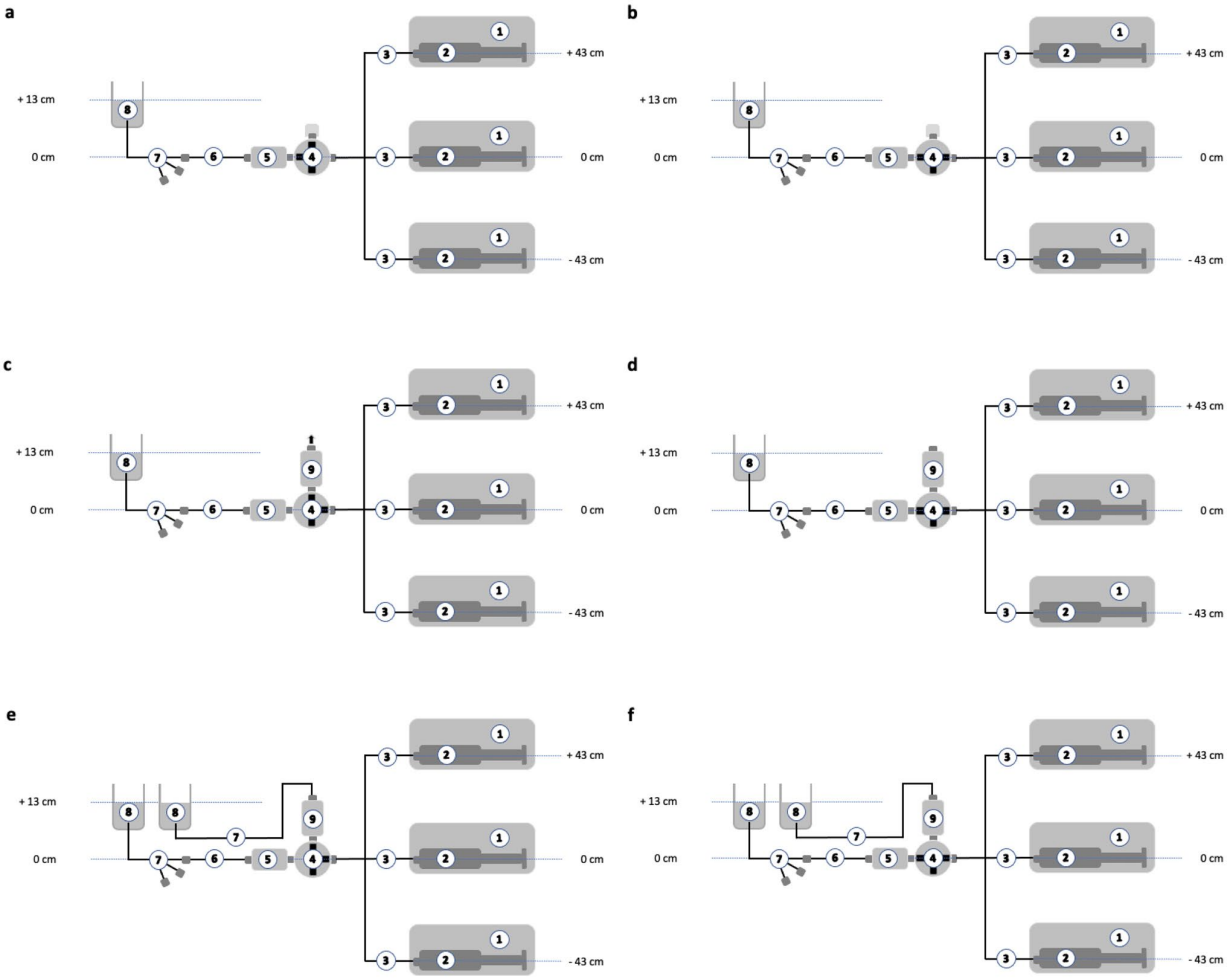
Schematic of the experimental in vitro setup for the measurement of fluid delivery after opening the infusion line to the central venous catheter and starting the pump at a set infusion rate of 1 mL/h without venting (Panel a and b), with ambient pressure venting (Panel c and d) and central venous pressure adjusted venting (Panel e and f) with the syringe infusion pump positioned at vertical positions levels of 0 cm, − 43 cm and + 43 cm. (1) Syringe pump; (2) 50 mL infusion syringe; (3) 2 m infusion line; (4) three-way stopcock; (5) primary liquid flow sensor; (6) 50 cm infusion line; (7) pediatric 5.5 Fr. three-lumen central venous catheter; (8) fluid chamber filled with distilled water and placed with the fluid surface level 13 cm above syringe and sensor outlet level; (9) secondary liquid flow sensor

The syringe pump loaded with a 50 mL syringe linked to a 2 m primed infusion line was placed at 43 cm below, at 43 cm above or at zero level, purged with a 1 mL fluid bolus and 1 min later connected to the closed infusion port of a three-way stopcock (Fig. 1a). The 1 mL fluid bolus was administered to eliminate mechanical gaps between the syringe and the pump for improved start-up performance [2]. Then, fluid flow measurement was initiated, the three-way stopcock opened and the pump immediately started (Fig. 1b).

#### Start-up with ambient pressure venting

The syringe pump loaded with a 50 mL infusion syringe linked to a 2 m primed infusion line was placed at 43 cm below, at 43 cm above or at zero level, purged with a 1 mL fluid bolus and connected to a three-way stopcock opened to ambient pressure to allow venting of fluid from the running syringe infusion pump assembly (Fig. 1c). Once a steady state flow was reached as confirmed by a second liquid flow sensor, fluid flow measurement of flow towards the central line was initiated, the vent was closed and fluid flow was directed to the central venous catheter (Fig. 1d).

#### Start-up with CVP-adjusted venting

The syringe pump loaded with a 50 mL infusion syringe linked to a 2 m primed infusion line was placed at 43 cm below, 43 cm above or at zero level, purged with a 1 mL fluid bolus and connected to a three-way stopcock opened to line with a pressure of 10 mmHg to allow venting of fluid from the running syringe infusion pump. The pressure of 10 mmHg used for venting was created by connecting the venting outlet with a fluid chamber filled with distilled water and placed with the fluid surface level 13 cm above the outlet level of the venting stopcock (Fig. 1e). Once steady state flow was reached as confirmed by a second liquid flow sensor, fluid flow measurement of flow towards the central line was initiated, the vent was closed and fluid flow directed to the central venous catheter (Fig. 1f).

Each measurement was repeated five times with two identical syringe infusion pumps (pump A/B). The different experimental setups were done in randomized order (in total 3 experimental setups × 5 repetitions × 2 pumps × 3 pump heights = 90 start-up runs) (www.random.org). Experiments were performed at a room temperature of 20.0 to 22.0 °C.

### Outcome parameters

The primary outcome parameters were infusion volumes delivered at 10, 60, 180 and 360 s after starting the pump, as well as the maximum backflow volume in case of retrograde fluid flow after opening the stopcock. Secondary outcome parameters were the time from starting the pump to flow inversion as well as the time required from starting the pump until the retrograde fluid volume was pushed back (zero drug delivery time). Further secondary outcome parameters included the time from starting the pump until steady state flow rates of ≥ 90% and ≥ 95% of the set flow rate were reached.

### Statistical analysis

Longitudinal analysis of delivered fluid over time was approached by means of hierarchical linear mixed effects model analysis. As independent variable the fixed effects time, modelled by means of a natural cubic spline with two degrees of freedom, setup and position were entered into the model, with a three-way interaction term. A natural cubic spline was chosen in order to enable a data-driven approximation of the expected biphasic deceleration/acceleration response of the infusion fluid after opening the stopcock. As random effects, intercepts for subjects nested in pumps were employed. P values were calculated using Satterthwaite’s method. In order to model maximal backflow volumes and zero drug delivery times, linear mixed-effects models with position and setup as fixed effects, including an interaction term, and pump as random effect, were employed. The intraclass correlation coefficient (ICC) was calculated to determine the data variance attributable to the pump specimen (A/B).

Statistical analysis was performed through a fully scripted data management pathway using the R environment for statistical computing version 4.2.3 (R Core Team, Vienna, Austria). Data are presented as means with 95% confidence intervals.

## Results

### Primary outcome parameters

**Fig. 2:**
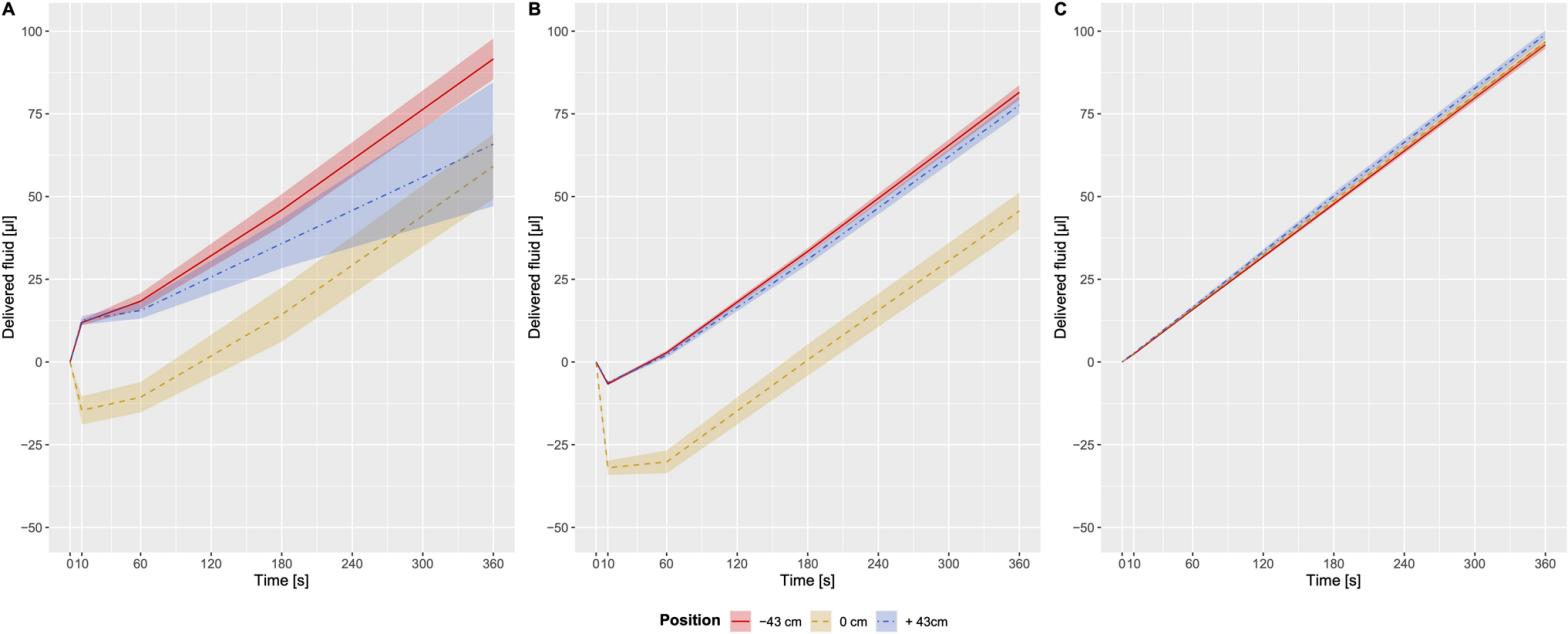
Delivered fluid volumes from the pump placed at vertical levels of 0 cm, − 43 cm and + 43 cm measured 10, 60, 180 and 360 s after opening the infusion line to the central venous catheter and immediately starting the pump at a set infusion rate of 1 mL/h with non-venting (**A**), with ambient pressure venting (**B**) and with central venous pressure adjusted venting (**C**). Estimated effects are presented as lines, 95% confidence intervals are presented as shaded colored areas. *CVP* central venous pressure

Infusion volumes delivered after opening the stopcock to the central venous catheter significantly varied among the three setups over time (p < 0.001) (Table 1, Fig. 2). The best percentual values of measured to expected (calculated) fluid delivery at 10, 60, 180 and 360 s after start-up were found for CVP-adjusted venting (87% to 100%) whereas the largest spread of values was found for ambient pressure venting (− 1151% to + 82%). Overall, 360 s after starting the pump 72% to 92% of expected fluid volumes were measured in the non-vented setup, 46% to 82% in the ambient pressure vented setup and 96% to 99% in the CVP-adjusted venting setup, depending on pump position (Table 1).

**Table 1:** Infusion volumes delivered with syringe pump start-up using the non-vented, ambient pressure vented and central venous pressure adjusted venting approach at a set flow rate of 1 mL/h

Experimental setup	Vertical pump position	Infusion volume delivered (µL) percentage of delivered versus calculated infusion volume (%)			
	cm	10 s	60 s	180 s	360 s
Non-vented start-up	+ 43	12.56 [11.29 to 13.83] 452%	15.60 [13.11 to 18.08] 94%	35.79 [28.36 to 43.22] 72%	73.16 [47.10 to 99.22] 72%
	0	− 14.62 [− 18.89 to − 10.35] − 526%	− 10.65 [− 15.22 to − 6.07] − 64%	14.32 [6.14 to 22.51] 29%	59.13 [49.37 to 68.89] 59%
	− 43	11.93 [11.08 to 12.79] 430%	18.39 [15.98 to 20.80] 110%	45.90 [41.07 to 50.73] 92%	91.60 [85.43 to 97.77] 92%
Ambient pressure vented start-up	+ 43	− 6.23 [− 6.53 to − 5.92] − 224%	2.10 [1.26 to 2.94] − 13%	30.99 [29.41 to 32.57] 62%	77.66 [75.05 to 80.27] 78%
	0	− 31.97 [− 34.15 to − 29.79] − 1151%	− 30.19 [− 33.63 to − 26.75] − 181%	0.62 [− 4.13 to 5.36] 1%	45.66 [40.08 to 51.23] 46%
	− 43	− 6.63 [− 7.08 to − 6.18] − 239%	2.83 [2.18 to 3.47] 17%	33.42 [32.41 to 34.43] 67%	81.52 [79.32 to 83.71] 82%
CVP-adjusted vented start-up	+ 43	2.72 [2.61 to 2.84] 98%	16.54 [16.12 to 16.96] 100%	50.09 [48.95 to 51.23] 100%	98.98 [97.71 to 100.25] 99%
	0	2.58 [2.48 to 2.67] 93%	16.22 [15.87 to 16.57] 97%	48.72 [47.99 to 49.45] 97%	96.75 [95.50 to 97.99] 97%
	− 43	2.42 [2.22 to 2.61] 87%	15.86 [15.63 to 16.09] 95%	47.76 [47.05 to 48.47] 96%	95.86 [97.68 to 97.04] 96%

Data presented as mean [CI 95%] and percentage of measured versus calculated infusion volume (%)

Start-up fluid delivery using the CVP-adjusted venting approach was similar among the three vertical pump positions tested (p = 0.485) while there were considerable differences in the non-vented and in the ambient pressure vented setup depending on pump position (p < 0.001 for both setups).

Backflow volumes were observed in the non-vented setup with the pump placed at zero level and in the ambient pressure vented setup with all three vertical pump positions (Tables 1 and 2). The most substantial backflow volumes with the pump at zero level (heart level) were − 13.82 (− 14.95 to − 12.69) µL and − 36.24 (− 37.47 to − 35.02) µL for non-vented and ambient pressure vented setups, respectively. Remarkably, no backflow was detected when employing the CVP-adjusted venting strategy. Differences in maximal backflow volumes among the three vertical pump positions were found in the non-vented and in the ambient pressure vented setup (p < 0.001).

**Table 2:** Maximum backflow volumes, times to flow inversion and zero drug delivery times using the non-vented, ambient pressure vented and central venous pressure adjusted venting approach at a set flow rate of 1 mL/h

Experimental setup	Vertical pump position cm	Maximum backflow volume(µL)	Time to flow inversion (min)	Zero drug delivery time (min)
Non-vented start-up	+ 43	0 [0 to 0]	Not applicable	Not applicable
	0	− 13.82 [− 14.95 to -12.69]	0.44 [0.39 to 0.48]	2.10 [1.88 to 2.32]
	− 43	0 [0 to 0]	Not applicable	Not applicable
Ambient pressure vented start-up	+ 43	− 6.43 [− 6.54 to − 6.32]	0.11 [0.10 to 0.12]	0.76 [0.70 to 0.82]
	0	− 36.24 [− 37.47 to − 35.02]	0.34 [0.33 to 0.35]	3.06 [2.94 to 3.17]
	− 43	− 6.84 [− 7.01 to − 6.67]	0.10 [0.10 to 0.11]	0.80 [0.78 to 0.82]
CVP-adjusted vented start-up	+ 43	0 [0 to 0]	Not applicable	Not applicable
	0	0 [0 to 0]	Not applicable	Not applicable
	− 43	0 [0 to 0]	Not applicable	Not applicable

Data presented as mean [CI 95%]

### Secondary outcome parameters

The times to flow inversion as well as the time until the backflow volume was pushed out of the syringe pump assembly (zero drug delivery time) are shown in Table 2. Mean start-up times until 90% steady state flow rate of set flow rate was reached were highest (2.82–4.59 min) in the non-vented setup, about half (1.69 to 2.56 min) in the ambient pressure vented setup and very short (0.08 to 0.1 min) in the CVP-adjusted venting approach for all three vertical pump positions (Table 3).

**Table 3:** Start-up times using the non-vented, ambient pressure vented and central venous pressure adjusted venting approach at a set flow rate of 1 mL/h

Experimental setup	Vertical pump position	Start-up time (min)	
	cm	> 90% SSFR	> 95% SSFR
Non-vented start-up	+ 43	4.59 [3.67 to 5.51]	5.94 [4.73 to 7.16]
	0	4.26 [3.43 to 5.09]	6.62 [5.11 to 8.13]
	− 43	2.82 [2.24 to 3.41]	5.81 [4.23 to 7.39]
Ambient pressure vented start-up	+ 43	2.56 [2.29 to 2.83]	4.64 [4.14 to 5.15]
	0	2.05 [1.87 to 2.24]	3.28 [2.88 to 3.67]
	− 43	1.69 [1.56 to 1.82]	3.74 [2.77 to 4.71]
CVP-adjusted venting start-up	+ 43	0.08 [0.08 to 0.08]	0.13 [0.11 to 0.15]
	0	0.09 [0.09 to 0.09]	0.19 [0.16 to 0.22]
	− 43	0.10 [0.09 to 0.11]	0.20 [0.16 to 0.24]

Data presented as mean [CI 95%]

*CVP* central venous pressure, *SSFR* steady state flow rate

The intraclass correlation coefficient (ICC) between both pumps was 0.005, indicating a very low variance attributable to the pump specimen.

## Discussion

The current in vitro assessment compared start-up fluid delivery after opening the three-way stopcock to a CVP of 10 mmHg immediately followed by starting the pump when using the non-vented, ambient pressure vented and CVP-adjusted vented approach.

Our main observation was the superior performance of the CVP-adjusted venting method. Unlike its counterparts, it efficiently inhibited pressure-induced volume shifts after opening the stopcock and almost nullified any lags in start-up fluid delivery. Additionally, this method effectively adjusted for varied vertical pump placements upon opening the stopcock and activating the pump.

Measured start-up fluid delivery using the non-vented approach is in agreement with earlier published results and also confirms the unreliable course of fluid delivery with the pump placed above and below zero level compared to the pump placed at heart level [9].

Using ambient pressure venting with the pump placed at zero level, higher backflow volumes and shorter times until steady state flow rate were observed than with non-vented start-up. This is in line with with recently published data [8]. The observed higher backflow volume measured with ambient pressure venting is explained by the higher pressure gradient from “CVP to infusion line” compared to the non-vented approach. This is because without venting, screwing the distal end of the infusion line into the infusion port of the closed three-way stopcock pressurizes fluid in the syringe infusion pump assembly, while in the vented setup free drainage of fluid to ambient pressure prevents such a pressure increase [8, 9].

A new secondary finding was that in contrast to non-venting, start-up fluid delivery using ambient pressure venting with the pump placed above or below zero level resulted in similar small backflow volumes after opening the stopcock. These are again related to the higher pressure gradients with ambient pressure venting and led to the largest spread of values found among the three setups tested.

CVP-adjusted venting, i.e. prerunning the syringe infusion pump assembly to a pressure equal to CVP allowed start-up fluid delivery with minimal deviations from set flow rate (0% to 5%) as measured at 60, 180 and 360 s after directing the flow to the central venous catheters. No anterograde or retrograde bolus volumes were detectable and start-up times to establish 90% and 95% steady state flow rate were very short, independent of vertical pump position.

The findings of this study have important clinical implications. Fast onset of precise drug delivery is crucial when initiating cardiovascular drugs in hemodynamically unstable patients. Prolonged lag times in drug delivery may result in persistent hemodynamic instability or cardiac arrest. Even when using very low flow rates, the CVP-adjusted venting allows for a very precise fluid delivery with very short start-up times while avoiding backflow volumes, regardless of pump position.

Administering short half-life vasoactive drugs mandates consistent and precise delivery. With syringes demanding periodic replacements, either due to nearing depletion or based on institutional policy, inconsistencies during these changeovers can induce blood pressure volatility [10]. Dramatic shifts in blood pressure, stemming from abrupt fluid surges or backflows, have been observed post stopcock opening and pump activation [11]. Current evidence highlights that, irrespective of the changeover methodology, a significant fraction of critically ill patients on norepinephrine or epinephrine face hemodynamic instabilities during these procedures [3, 10]. Notably, the CVP level and vertical pump position have been identified as culprits for fluid inconsistencies during pump changeovers [9, 12–14]. In light of our findings, CVP-adjusted venting emerges as a promising solution to counter these challenges in the foreseeable future [3, 10, 11]. The act of adjusting venting outlet pressure to CVP certainly needs more comfortable technical solutions than using a reservoir filled with water adjusted a specific level above heart level. Ideally, an adjustable pressure valve at the venting outlet positioned at heart level would allow for an easy venting adjusted to patient’s current CVP before a syringe pump changeover is performed. A more simplified technique would involve employing a check valve with an opening pressure of 10 mmHg at the venting outlet. This method would serve to mitigate extensive pressure disparities between the CVP and infusion line pressure [15].

Besides venting pressure management, venting fluid from the running syringe pump demands a more sterile approach in clinical practice, such as a waste collecting bag. Moreover, the assembly of several stopcocks for infusion venting poses challenges. These encompass increased workload, complications in air evacuation from dead spaces, potential unsterile fluid wastage, and operational complexities, such as the potential for accidental line occlusion. A simplified, pre-assembled device incorporating both a pressure valve and a waste collection compartment would be desirable.

The current study used solely one level of flow rate. It is conceivable that lower or higher flow rates may modify the presented results. Furthermore, only one brand and size of syringes were included in the study and it is possible that varying compliances of different syringe infusion pump assemblies may affect pressure-induced fluid shifts as well as start-up times [16].

## Conclusion

CVP-adjusted venting almost nullifies start-up times to steady state flow rate using microinfusions and eliminates pressure-induced fluid shifts after opening the stopcock. This new approach is promising for the precise delivery of vasoactive and inotropic drugs in critical care when starting a new infusion or during syringe pump changeover.

## Declarations

### Humans or animals

The study did not include humans or animals so there was no need for ethical approval. No data or picutres of humans or animals are included in the manuscript. Only technical equiment was tested in the lab.
